# LRH‐1 activation alleviates diabetes‐induced podocyte injury by promoting GLS2‐mediated glutaminolysis

**DOI:** 10.1111/cpr.13479

**Published:** 2023-04-13

**Authors:** Jijia Hu, Zongwei Zhang, Hongtu Hu, Keju Yang, Zijing Zhu, Qian Yang, Wei Liang

**Affiliations:** ^1^ Division of Nephrology Renmin Hospital of Wuhan University Wuhan Hubei China; ^2^ Nephrology and Urology Research Institute of Wuhan University Wuhan Hubei China; ^3^ The First College of Clinical Medical Science, China Three Gorges University Yichang Hubei China

## Abstract

Alteration of metabolic phenotype in podocytes directly contributes to the development of albuminuria and renal injury in conditions of diabetic kidney disease (DKD). This study aimed to identify and evaluate liver receptor homologue‐1 (LRH‐1) as a possible therapeutic target that alleviates glutamine (Gln) metabolism disorders and mitigates podocyte injury in DKD. Metabolomic and transcriptomic analyses were performed to characterize amino acid metabolism changes in the glomeruli of diabetic mice. Next, Western blotting, immunohistochemistry assays, and immunofluorescence staining were used to detect the expression of different genes in vitro and in vivo. Furthermore, Gln and glutamate (Glu) content as well as ATP generation were examined. A decrease in LRH‐1 and glutaminase 2 (GLS2) expression was detected in diabetic podocytes. Conversely, the administration of LRH‐1 agonist (DLPC) upregulated the expression of GLS2 and promoted glutaminolysis, with an improvement in mitochondrial dysfunction and less apoptosis in podocytes compared to those in vehicle‐treated db/db mice. Our study indicates the essential role of LRH‐1 in governing the Gln metabolism of podocytes, targeting LRH‐1 could restore podocytes from diabetes‐induced disturbed glutaminolysis in mitochondria.

## Graphical abstract

Impairment of glutamine degradation and utilization in podocytes under diabetic kidney disease: LRH‐1 positively regulates GlS2‐mediated glutaminolysis, and downregulation of LRH‐1 expression contributes to diabetes‐induced disturbance of energy metabolism and injury in podocytes.

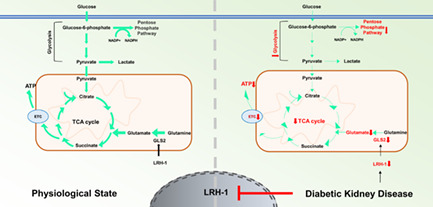

## 1 INTRODUCTION

Diabetic kidney disease (DKD) is the common cause of end‐stage renal disease (ESRD) worldwide. Current interventions such as hypoglycemic agents, renin‐angiotensin‐aldosterone system (RAAS) blockers, and sodium‐glucose cotransporter 2 (SGLT2) inhibitors are not able to completely prevent the occurrence and progression of DKD.^1^, ^2^ Hence, a revelation of the underlying pathogenesis of DKD is fundamental for finding therapeutic targets.

Podocytes are highly specialized and terminally differentiated visceral epithelial cells essential for maintaining the glomerular filtration barrier (GBM).^3^ Podocyte injury has been considered a critical process for the initiation and progression of DKD.^4^ Recently, metabolomics from our studies has shown that impaired amino acid metabolism is associated with the development of DKD.^5^, ^6^ Notably, increasing evidence has shed more light on the involvement of glutamine (Gln) in renal metabolism.^7^, ^8^ As the most abundant and versatile amino acid in the human body, Gln participates in energy generation, metabolism homeostasis, cell proliferation, and apoptosis.^9^, ^10^ Interestingly, several studies have reported that podocytes rely on Gln for maintaining cellular structure and function, and Gln supplementation could reduce proteinuria and ameliorate glomerular injury in LPS‐treated mice.^11^, ^12^ Thus, the stepwise changes of Gln metabolism may be involved in DKD‐induced podocyte injury, but the mechanism is yet to be clarified.

Liver receptor homologue‐1 (LRH‐1), also known as nuclear receptor subfamily 5 group A member 2 (NR5A2), belongs to an orphan nuclear receptor, which is involved in lipid, glucose, and amino acid metabolism.^13^, ^14^ Although studies have revealed that LRH‐1 regulates hepatocyte metabolism and affects the survival or death of cancer cells by participating in mitochondrial Gln metabolism,^15^ it remains unknown whether LRH‐1 is involved in podocyte Gln metabolism and its effect on DKD‐induced podocyte injury has still not been fully clarified. Here, we identified a novel role of LRH‐1 in alleviating diabetes‐induced podocyte injury via enhancing glutaminases 2 (GLS2)‐dependent Gln mobilization and utilization in mitochondria. These results suggest that LRH‐1 might serve as a new therapeutic target for podocyte injury in DKD.

## 2 MATERIALS AND METHODS

### 2.1 Antibodies and reagents

Rabbit anti‐LRH‐1 antibodies were obtained from Proteintech (Wuhan, China, 22460‐1‐AP); mouse anti‐WT1 antibodies were purchased from Novus (Colorado, USA, NB110‐60011); rabbit anti‐GLS2 antibodies were purchased from Abcam (Cambridge, UK, ab113509), guinea pig anti‐Synaptopodin antibody was obtained from Progen Biotechnik (Heidelberg, Germany, GP94); anti‐GAPDH mouse monoclonal antibody, fluorescent secondary antibodies were purchased from Antgene (Wuhan, China, ANT324). 1,2‐dilauroyl‐sn‐glycerol‐3‐phosphocholine (DLPC) was purchased from MedChemExpress (Shanghai, China, HY‐107737), dissolved in glycolic acid (GC) at 23°C, and kept at −20°C.

### 2.2 Animal study

All animal protocols were approved by the Animal Care Committee of Renmin Hospital of Wuhan University. Male mice (6 weeks) on C57BL/KsJ‐db/db (db/db, *n* = 12) and C57BL/KsJ‐db/m (db/m, *n* = 12) background was purchased from Cavens Laboratory Animal Co., LTD, Changzhou, China. After adaptive feeding for 2 weeks, all mice were randomly divided into indicated groups using the random number table method. For the DKD model, mice were raised to 16 weeks from the onset of diabetes (six mice in each group). DKD was defined as diabetes with the presence of microalbuminuria or proteinuria, impaired renal filtration function, manifested as elevated urinary albumin‐to‐creatinine ratio (UACR), the induction success rate was 100%. For LRH‐1 agonist treatment, the db/m and db/db mice were divided into four groups (db/m control, db/db control, db/m + DLPC, db/db + DLPC, three mice in each group), mice were treated daily by oral gavage with DLPC (100 mg/kg) or the solvent vehicle. During the above treatment period, none of the mice died. On the last day of the sixteenth week, the animals were placed in metabolic chambers for 24‐h urine collection, and then sacrificed under anaesthesia, the kidneys harvested were collected for subsequent examinations.

### 2.3 Cell cultures and treatments

Conditionally immortalized human podocytes (HPCs) cell line was obtained from Dr. Moin A. Saleem (Academic Renal Unit, Southmead Hospital, Bristol, UK). HPCs were grown as described previously.^16^ Briefly, HPCs were cultured in RPMI‐1640 basal medium (glucose 5.5 mM, HyClone, USA) containing 10% heat‐inactivated fetal bovine serum (FBS; Gibco, USA), penicillin G (100 IU/mL), streptomycin (100 mg/mL) and 1× insulin‐transferrin‐selenium (ITS; Invitrogen, USA) at 33°C for proliferation; then they were thermoswitched to 37°C for 10–14 days without ITS to induce differentiation. All experiments were performed with differentiated cells. For high glucose (HG) stimulation, podocytes were incubated with a high concentration (30 mM) of glucose for 24 h, and mannitol (30 mM) was used as an osmotic control. For plasmid transfection, podocytes were transfected with the pEnCMV‐Nr5a2 (LRH‐1 pcDNA) plasmid and control plasmid (Miaolingbio, China) using Lipofectamine 3000 Transfection Kit (Invitrogen, USA) according to the manufacturer's instructions. For interference treatment, the small interfering RNA (siRNA) targeting GLS2 (5′‐ATCAAGATGGACTGTAA‐CAAA‐3′) was transfected into podocytes with HiPerFect (Qiagen, Germany) according to the manufacturer's instructions.

### 2.4 Apoptosis assay

Apoptosis in cultured cells was determined by flow cytometry (BD Biosciences, USA) using a PE‐annexin V with 7‐AAD double staining kit according to the manufacturer's statement (BioLegend, USA). The percentage of cell apoptosis for each sample was calculated using FlowJo software, apoptosis rate = percentage of early apoptosis (Q3) + percentage of advanced apoptosis (Q2). Apoptosis in renal tissues was determined by TUNEL staining according to the manufacturer's protocol (Roche, Switzerland). The TUNEL‐positive cells in the glomeruli were observed and analysed using a microscope (Olympus Co., Japan).

### 2.5 Isolation of glomeruli

The glomeruli were isolated using the sieve method. Briefly, renal cortices from mice were minced and digested with collagenase. The digested tissue was then gently pressed through 100‐, 70‐, and 40‐ mm mesh sieves, followed by intermittent ice‐cold sterile PBS flushing. Glomeruli‐rich preparation on the 40‐ mm strainer was dissolved in PBS, and then the suspension was transferred into 15 mL centrifuge tubes. After centrifugation, glomeruli were collected into cryopreservation tubes. The entire process was performed on ice except for collagenase digestion, which was performed in a warm chamber.

### 2.6 Metabolomic analyses

Glomeruli were collected, and the metabolomic study was performed by Applied Protein Technology Co., Ltd. (Shanghai, China). Three independent replicate samples were analysed from each group.

### 2.7 RNA sequencing and transcriptomic analyses

Glomeruli were collected, and transcriptome sequencing was performed by Myhalic Biotechnological Co., Ltd. (Wuhan, China). Three independent replicate samples were analysed from each group. Data preprocessing and differentially expressed genes (DEGs) analysis were performed using R (version 4.0.0; MathSoft, Inc.).

### 2.8 Immunofluorescence assay

The frozen kidney sections or cell samples were fixed, then blocked and incubated with primary antibodies (LRH‐1: 1:100, GLS2: 1:100) overnight at 4°C, followed by incubation with fluorescent secondary antibodies at 37°C for 90 min in the dark. The nuclei were counterstained with 4, 6‐diamidino‐2‐phenylindole (DAPI, Antgene, China) for 5 min. All microscopic images were detected by confocal fluorescence microscopy (Olympus, Japan).

### 2.9 Immunohistochemical assay

Renal tissues from mice were embedded into paraffin, and 4‐μm tissue sections were prepared. Tissue sections were subjected to deparaffinized, rehydration, antigen retrieval, and blocking. Subsequently, sections were incubated with primary antibodies overnight at 4°C. Then, the sections were incubated with HRP‐conjugated secondary antibody for 30 min. After washing, samples were stained with diaminobenzidine for 5 min and counterstained in haematoxylin. Slides were examined by microscope (Olympus, Japan).

### 2.10 Periodic acid‐schiff (PAS) staining

PAS staining was performed with Periodic Acid Schiff (PAS) Stain Kit (Servicebio, China), according to the manufacturer's instructions. Slides were examined by light microscope (Olympus, Japan). Mesangial matrix expansion was defined as the increased amounts of PAS‐positive material in the mesangial region and evaluated by the glomerular sclerosis index.^17^ Twenty glomeruli per mouse were assessed.

### 2.11 Western immunoblotting

Cells or tissues (isolated glomeruli) were collected with RIPA buffer, and protein concentration was determined by bicinchoninic acid (BCA) assays (Beyotime, China). Equal amounts of protein (30 μg per lane) were separated by SDS‐PAGE and then transferred to PVDF membranes (Millipore Corp, USA). The membranes were then incubated with a primary antibody (LRH‐1: 1:500, GLS2: 1:1000, GAPDH: 1:5000) in 5% milk overnight at 4°C. Next, the membranes were labelled with an Alexa Fluor 680/790‐labelled (1:10,000, LI‐COR Biosciences, USA) goat anti‐mouse/goat anti‐rabbit secondary antibody, followed by scanning via an LI‐COR Odyssey Infrared Imaging System.

### 2.12 Glutamine and glutamate detection

Commercial detection kits, including a glutamine assay kit and a glutamate assay kit (Biovision, Milpitas, CA, USA), were utilized to detect the content of glutamine and glutamate in HPCs according to matched protocols.

### 2.13 ATP production

After treatment, the intracellular ATP content was assessed using a luciferase ATP detection assay kit (Beyotime, China) according to the manufacturer's protocol. Briefly, the cells were harvested and lysed. Then, the protein content of the supernatant was determined with a BCA protein kit (Beyotime, China), and the protein supernatant was mixed with an ATP detection solution. Finally, the luminescence of each sample was measured by a fluorescence microplate reader.

### 2.14 Statistics

At least 3 experiments were conducted, and representative experiments are shown. Quantitative data were expressed as means ± SEM, and statistical analyses were performed using GraphPad Prism 7 software. Continuous variables for two groups were compared using Student *t*‐tests. Continuous variables for more than two groups were compared using a 1‐way ANOVA test. Statistical analyses of the data were performed using GraphPad Prism (Version 7.0, CA). A *p* value less than 0.05 was considered statistically significant.

## 3 RESULTS

### 3.1 Renal morphology and amino acid metabolism in the glomeruli of db/db mice

**FIGURE 1:**
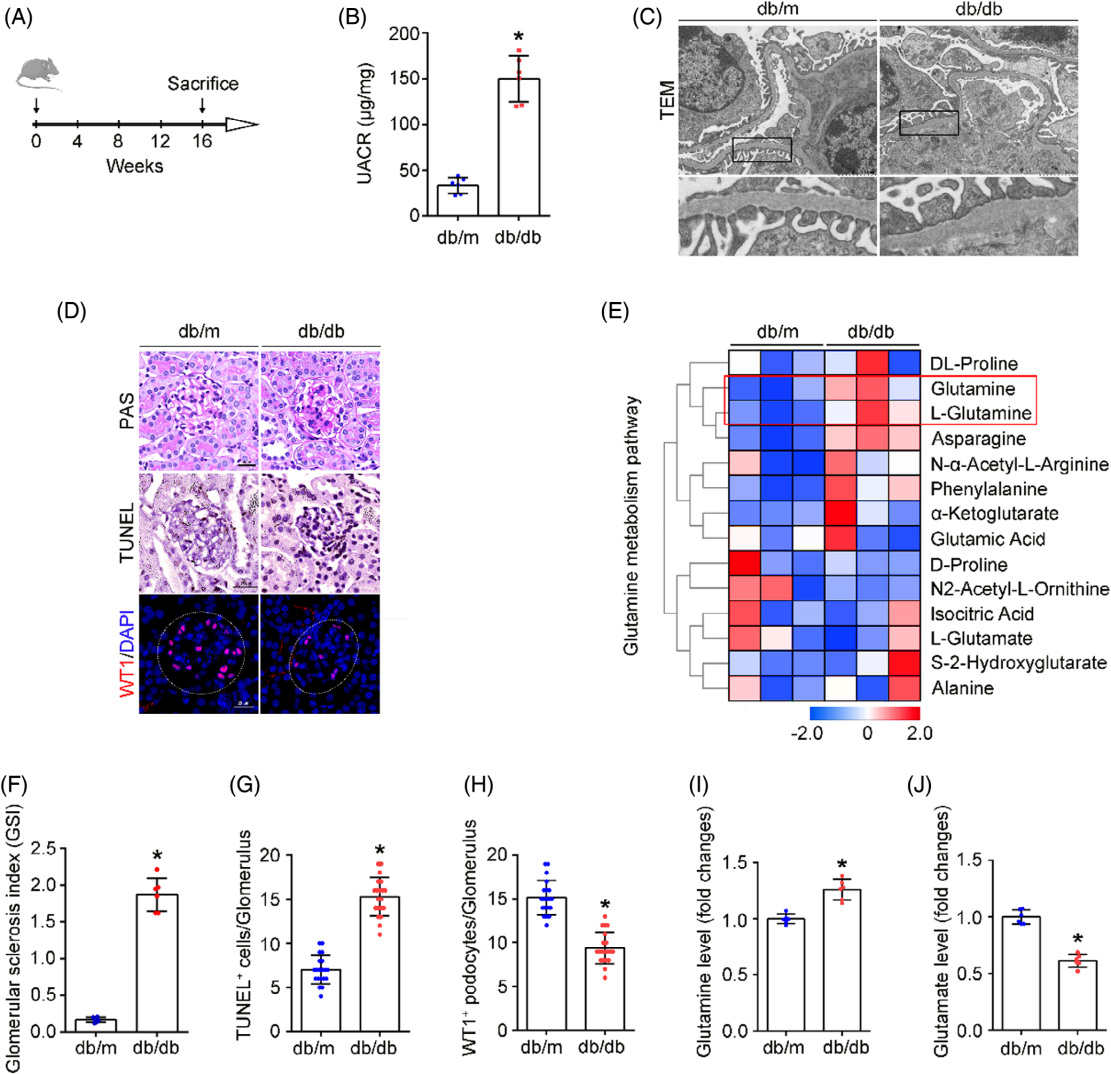
Activation of glutamine metabolism pathway in glomeruli from DKD mice. (A) Different groups of mice were sacrificed at 16 weeks from the onset of this study. (B) Urinary albumin‐to‐creatinine ratio (UACR) in different groups of mice. *p < 0.05, *n* = 6. (C) Representative transmission electron microscopy (TEM) pictures showing podocyte foot process effacement and glomerular basement membrane (GBM) thickness in mice with DKD. Scale bars: 2 μm. (D) Representative periodic acid–Schiff (PAS) staining, Terminal labelling (TUNEL) staining, and immunofluorescence staining (WT1‐positive podocytes) in glomerulus from renal tissues. Scale bar: 25 μm. (E) Heatmap of the metabolite changes in glomeruli from different groups of mice. (F) Glomerular sclerosis index of the PAS staining, *p < 0.05 (*n* = 6). (G) Quantitative analyses of TUNEL‐staining‐positive cell per glomerulus in renal tissues. *p < 0.05 (*n* = 20). (H) Quantitative analyses of WT1‐staining‐positive podocytes per glomerulus in renal tissues. *p < 0.05 (*n* = 20). (I, J) The relative glomerular content of Gln and Glu. *p < 0.05 (*n* = 6).

To elucidate the effect of diabetes on glomerular amino acid metabolism, the db/db mice, a well‐established diabetic model with progressive kidney injury, were raised (Figure 1A). Regarding renal phenotypic changes, db/db mice presented an increased urinary albumin‐to‐creatinine ratio (UACR) (Figure 1B). Morphological changes were observed by transmission electron microscopy (TEM) and PAS staining (Figure 1C,D,F). TEM exhibited obvious podocyte foot process effacement, dilated mesangial matrix, and significant glomerulosclerosis in db/db mice. Additionally, increased TUNEL staining apoptotic glomerular cells and decreased Wilms tumour 1‐positive (WT1‐positive) podocyte number were observed in db/db mice (Figure 1D,G,H). Subsequently, isolated glomeruli were subjected to metabolomic analyses (Figure 1E), as revealed inhibited glutaminolysis under diabetic conditions with enriched Gln/L‐Gln in db/db mice than in db/m mice. To further confirm the pattern of Gln catabolism, the content of Gln and Glu in the glomeruli of different groups of mice was quantified (Figure 1I,J). In line with the metabolomic results, elevated Gln was detected in the glomeruli of db/db mice compared with that in db/m mice, while Glu was in an opposite pattern with Gln in the glomeruli. Collectively, these data indicate the presence of obstructed glutaminolysis in glomeruli from mice with DKD.

### 3.2 Profiling of gene expression involved in Gln metabolism in the glomeruli of db/db mice

**FIGURE 2:**
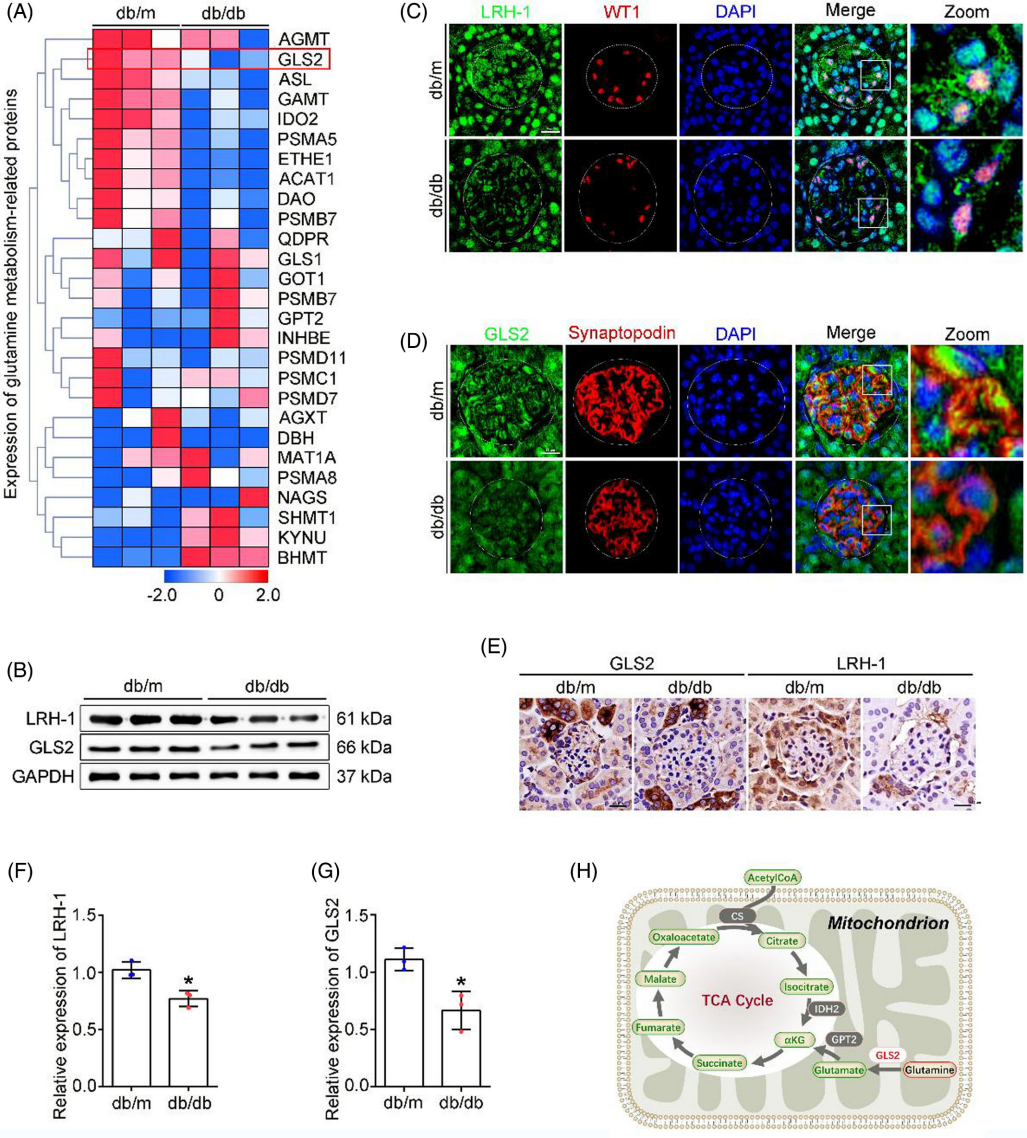
Changes in critical enzymes of Gln catabolism in the glomerular podocytes of db/db mice. (A) Heatmap showing the changes in critical metabolic enzymes of glutaminolysis and amino acid catabolism. (B) Western blot assay showing expression of glomerular LRH‐1 and GLS2. *p < 0.05 (*n* = 3). (C, D) Representative immunofluorescence staining of LRH‐1 and GLS2 with WT1/Synaptopodin‐positive podocytes in glomerulus from renal tissues among different groups. Scale bar: 25 μm. (E) Representative immunohistochemical staining images showing the reduction of LRH‐1 and GLS2 in glomerulus from renal tissues of db/db mice. Scale bar: 25 μm. (F, G) Quantitative analyses of Western blot assay. *p < 0.05 (*n* = 3). (H) Schematic diagram of mitochondrial Gln metabolism.

Glomeruli were isolated from kidneys, transcriptomic analyses were performed to systemically evaluate the changes in Gln metabolism‐related genes between db/db mice and db/m mice (Figure 2A). Intriguingly, the expression of GLS2, a key regulator of the first and rate‐limiting step of the glutaminolysis in mitochondria,^9^ was downregulated in the glomeruli of db/db mice. Given that LRH‐1 possesses distinct functions in the regulation of key enzymes in liver Gln metabolism,^15^ we assessed the expression of LRH‐1 and GLS2 in the renal tissues of mice. Western blot analysis showed that the expression of LRH‐1 and GLS2 was decreased in glomeruli from db/db mice (Figure 2B,F,G). Meanwhile, immunofluorescence co‐staining with podocyte marker (WT1, Synaptopodin) showed reduced fluorescent intensity (LRH‐1 and GLS2) in podocytes from db/db mice (Figure 2C,D), and immunohistochemistry staining revealed a similar pattern (Figure 2E). Given that GLS2 is the gatekeeper for the conversion of Gln to Glu, an indispensable process for Gln to enter the tricarboxylic acid (TCA) cycle (Figure 2H), these findings suggest that the inhibited GLS2‐derived glutaminolysis could promote the accumulation of Gln in glomeruli with DKD.

### 3.3 Effect of high glucose on podocyte Gln processing

**FIGURE 3:**
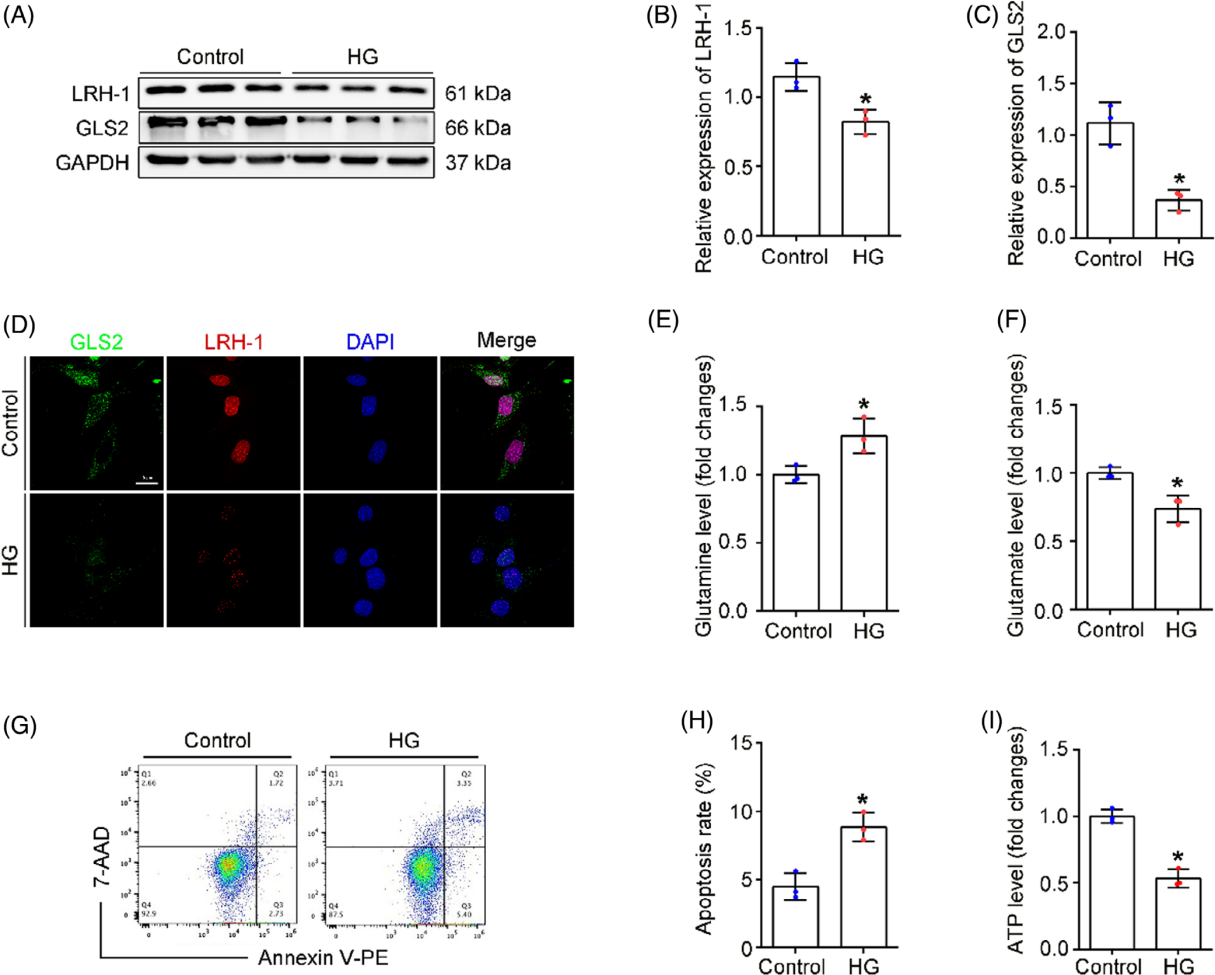
Changes in Gln catabolism of the cultured podocytes. (A) Western blot assay showing expression of LRH‐1 and GLS2 among different treatment groups. (B, C) Quantitative analyses of Western blot assay. *p < 0.05 (*n* = 3). (D) Representative immunofluorescence staining (LRH‐1, GLS2 with DAPI) in cultured podocytes. Scale bar: 10 μm. (E, F) The relative content of Gln and Glu in cultured podocytes. *p < 0.05 (*n* = 3). (G, H) The apoptosis rate of podocytes among different groups was determined by flow cytometry. *p < 0.05 (*n* = 3). (I) Quantitative analysis of ATP production in each group. *p < 0.05 (*n* = 3).

Next, the expression of LRH‐1 and GLS2 was evaluated in podocytes exposed to high glucose (HG) in vitro. In consist with in vivo studies, HG‐treated podocytes exhibited a significant decrease in LRH‐1 and GLS2, as shown by Western blot (Figure 3A–C) and immunofluorescence assays (Figure 3D). Since Gln was known as a fuel source of glutaminolysis to generate α‐ketoglutarate (α‐KG) and enter the TCA cycle,^18^ we next examined whether HG exposure affected glutaminolysis and the ability of energy production by mitochondria. As shown in Figure 3E,F, intracellular Gln levels were elevated in HG‐exposed podocytes, whereas the Glu content was decreased under HG exposure (Figure 3E,F). Consequently, decreased adenosine triphosphate (ATP) generation and elevated apoptosis rate were observed in HG‐exposed podocytes (Figure 3G–I), suggesting that HG exposure could disturb glutaminolysis activity in podocytes. Thus, the above results suggest that the decrease of LRH‐1 induced by HG likely leads to compromised glutaminolysis and failure of Gln utilization in the mitochondria of podocytes.

### 3.4 The LRH‐1‐GLS2 axis promotes Gln catalyzation and utilization

**FIGURE 4:**
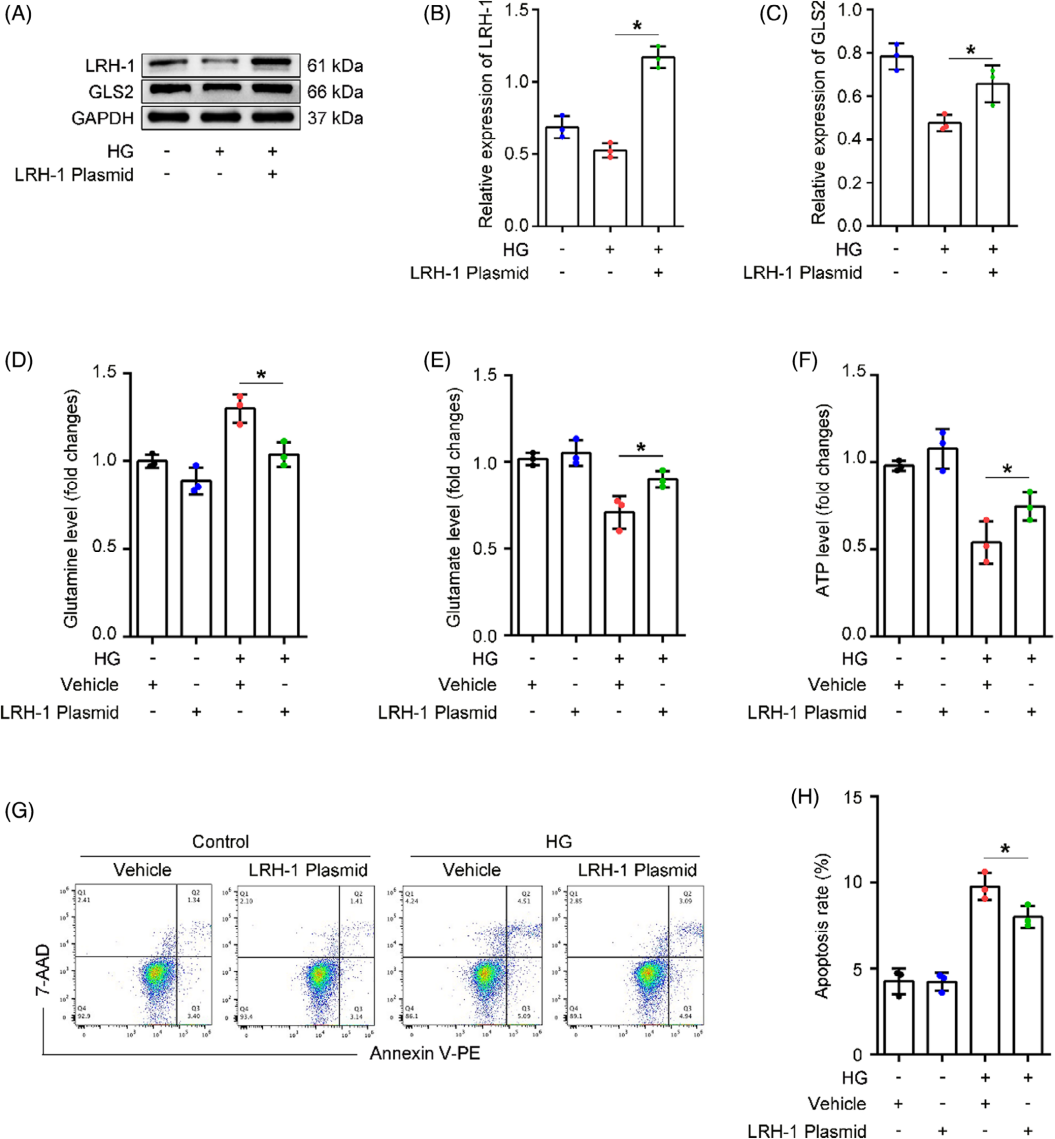
LRH‐1 overexpression alleviated HG‐induced compromised glutaminolysis, and mitochondrial Gln utilization. HPCs were transfected with LRH‐1 pcDNA plasmid and then exposed to HG for 24 h. (A) Western blot assay showing expression of LRH‐1 and GLS2 among different groups. (B, C) Quantitative analyses of Western blot assay. *p < 0.05 (*n* = 3). (D, E) The relative content of podocyte Gln and Glu among different groups. *p < 0.05 (*n* = 3). (F) Quantitative analysis of ATP production in each group. *p < 0.05 (*n* = 3). (G, H) Flow cytometry analysis of podocyte apoptosis among different groups. *p < 0.05 (*n* = 3).

To ascertain whether LRH‐1 was involved in Gln catabolism, podocytes were transfected with LRH‐1 pcDNA recombinant plasmid or control plasmid (Figure S1) and then incubated with HG for 24 h. Western blot analysis showed that LRH‐1 overexpression significantly restored the diminished expression of GLS2 induced by HG (Figure 4A–C). In addition, LRH‐1 overexpression promoted Gln catabolism, evidenced by an elevated level of cytoplasmic Glu even in the presence of HG exposure (Figure 4D,E). Consequently, the HG‐induced decrease of ATP production in podocytes was rescued by LRH‐1 overexpression (Figure 4F). Moreover, the cell apoptosis rate was slightly reduced in podocytes transfected with LRH‐1 plasmid under HG conditions (Figure 4G,H). To further distinguish the regulatory relationship between LRH1 and GLS2, the siRNA targeting GLS2 was used before HG stimulation. Meaningfully, podocytes transfected with GLS2 siRNA exhibited a significant decrease in GLS2 but not LRH‐1 expression compared with podocytes transfected with scrambled siRNA (Figure S2A–C). These results suggest that LRH‐1 participated in Gln utilization in a GLS2‐dependent manner.

### 3.5 DLPC administration enhances the expression of LRH‐1 and GLS2 in vivo

**FIGURE 5:**
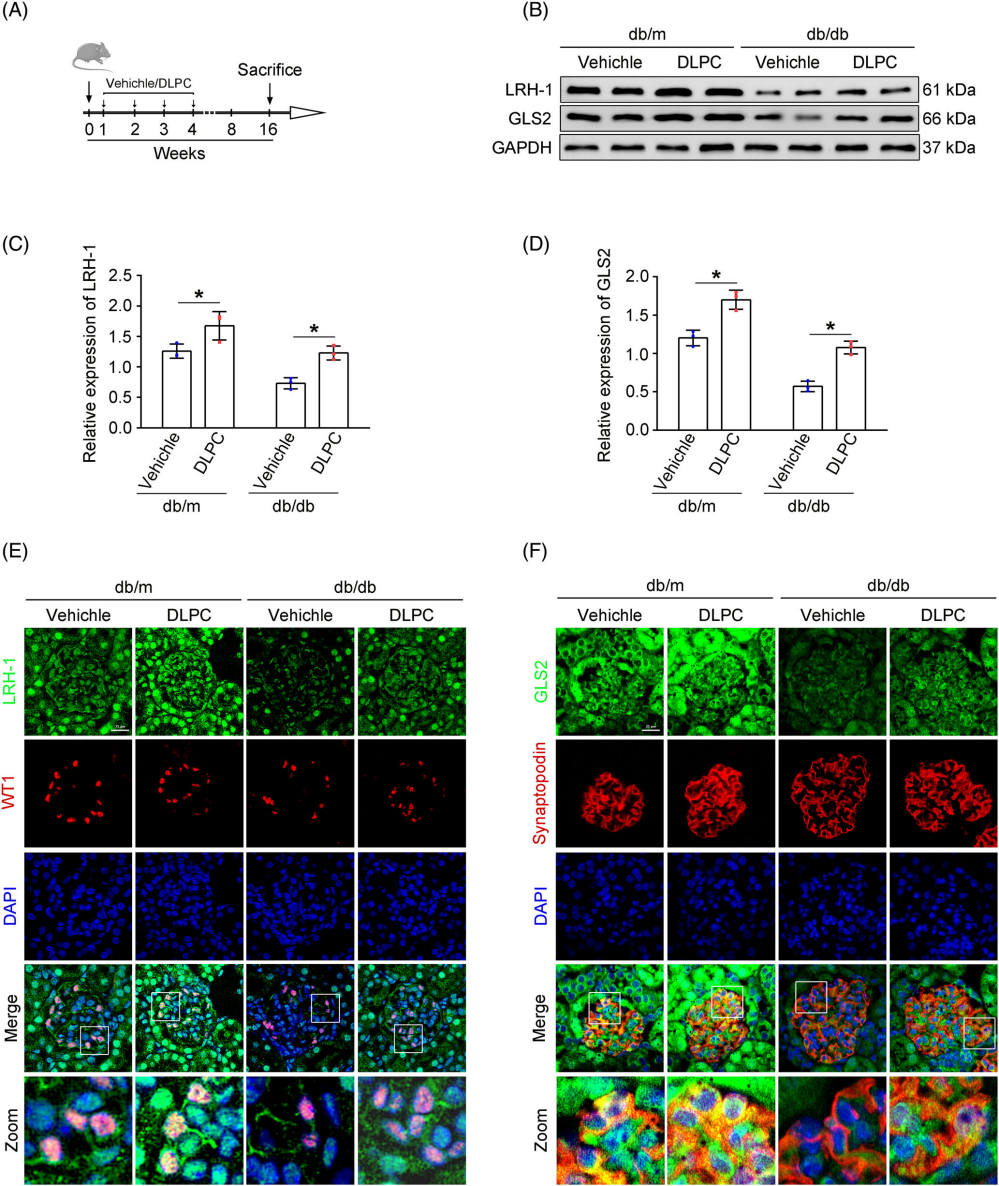
DLPC upregulated LRH‐1 and GLS2 expression. (A) Mice were treated daily by oral gavage with DLPC (100 mg/kg) or the solvent vehicle for 3 weeks and were sacrificed at 16 weeks after the onset of diabetes. (B) Western blot assay showing expression of LRH‐1 and GLS2 among different groups. (C, D) Quantitative analyses of Western blot assay. *p < 0.05 (*n* = 3). (E, F) Representative immunofluorescence staining of LRH‐1 and GLS2 with WT1/Synaptopodin‐positive podocytes in glomerulus from renal tissues among different groups. Scale bar: 25 μm.

Since the suspected link of LRH‐1 and GLS2‐induced glutaminolysis, we tested whether manipulation of the LRH‐1 signalling pathway by DLPC (a widely reported LRH‐1 agonist) could prevent the progression of DKD.^19^ With this, diabetic mice were administered daily by oral gavage with vehicle or DLPC for 3 weeks (Figure 5A). Then, the glomerular protein was prepared for Western blot analysis. As shown in Figure 5B–D, DLPC administration significantly induced upregulation of LRH‐1 and GLS2 in glomeruli from db/db mice, which was also evidenced by immunofluorescence co‐staining in podocytes when compared with those from vehicle‐administered db/db mice (Figure 5E,F).

### 3.6 DLPC administration alleviates renal damage in db/db mice

**FIGURE 6:**
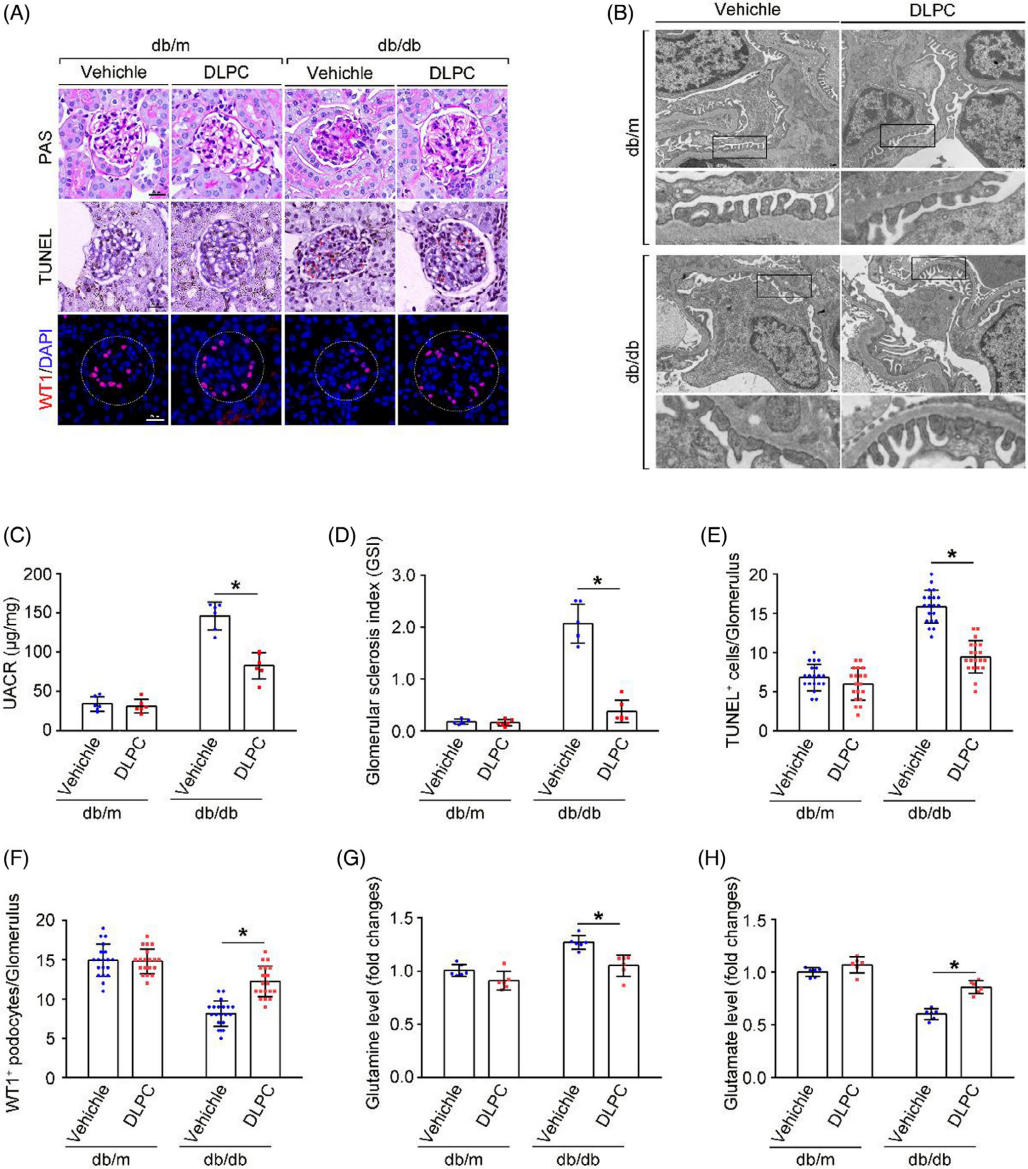
LRH‐1 activation attenuates podocyte injury and renal injury in DKD mice. (A) Representative periodic acid–Schiff (PAS) staining, Terminal labeling (TUNEL), and immunofluorescence staining (WT1‐positive podocytes) in glomerulus from renal tissues. Scale bar: 25 μm. (B) Representative transmission electron microscopy (TEM) pictures of glomerulus among different groups. Scale bars: 2 μm. (C) Urinary albumin‐to‐creatinine ratio (UACR) in different groups of mice. *p < 0.05, *n* = 6. (D) Glomerular sclerosis index of the PAS staining, *p < 0.05 (*n* = 6). (E) Quantitative analyses of TUNEL‐staining‐positive cell per glomerulus in renal tissues. *p < 0.05 (*n* = 20). (F) Quantitative analyses of WT1‐staining‐positive podocytes per glomerulus in renal tissues. *p < 0.05 (*n* = 20). (G, H) The relative glomerular content of Gln and Glu. *p < 0.05 (*n* = 6).

In addition to the promotion of Gln metabolism in podocytes by LRH‐1 activation, glomerular phenotypic alterations were evaluated. The UACR, the fusion of foot processes, glomerular lesions, and TUNEL staining apoptotic glomerular cells were alleviated by DLPC administration in db/db mice (Figure 6A–D). Intriguingly, the decreased number of podocytes in db/db mice was rescued by DLPC administration (Figure 6F). In addition, more conversion of Gln to Glu was detected in the glomeruli from DLPC‐administered db/db mice (Figure 6G,H). These results indicate that activation of LRH‐1 may ameliorate podocyte injury and shedding by enhancing GLS2‐mediated glutaminolysis and improving the ability of mitochondria energy metabolism in podocytes under DKD.

## 4 DISCUSSION

DKD has become a significant cause of chronic kidney disease (CKD) and end‐stage renal disease (ESRD).^20^ Emerging data from epidemiologic analyses, experimental studies, and clinical trials indicate DKD as a metabolic disorder, and the metabolic reprogramming of podocytes contributes to the pathogenesis of DKD.^21^, ^22^ Thus, targeting the metabolic pathways of podocytes is a promising therapeutic approach for DKD. In the current study, we revealed that LRH‐1 positively modulated the Gln metabolism in podocytes. Upregulation of LRH‐1 promoted the utilization of Gln by increasing GLS2 expression, thereby partially ameliorating mitochondrial disorders in podocytes under DKD.

Glucose and amino acids are essential nutrients that support biomass synthesis and energy generation in podocytes. However, amino acid metabolism has been less studied. While diabetes is characterized by hyperglycemia, nutrient metabolic pathways such as the TCA cycle are also profoundly perturbed.^23^ Other investigators and we recently reported that the insufficient energy supply caused by glycolytic pathway dysfunction in diabetic podocytes could be compensated by activating the TCA cycle processes with amino acids as metabolic substrates.^5^, ^6^ Indeed, Gln catabolism (glutaminolysis) is a critical component of the metabolic reprogramming, which is a potential source of carbon not only for ATP production via the TCA cycle, linked to oxidative phosphorylation (OXPHOS) in mitochondria, but likewise for the generation of precursors of nucleotides, amino acids, and some lipids.^24^ Previous evidence verified Gln as the alternative substrate of cells during tissue repair and regeneration for ATP production.^24^, ^25^ Intriguingly, a study has observed a physiological need for Gln in podocytes, and exogenous Gln supplementation improved the metabolic disorder and injury of podocytes.^12^ Notably, a meta‐analysis of prospective cohort studies has identified that higher plasma Gln was associated with lower type 2 diabetes risk.^26^ Studies also indicated that Gln supplementation was beneficial for preventing or delaying the onset of diabetes‐induced insulin resistance and cardiomyopathy.^27^, ^28^ In parallel, one of the most significant results of our study was the discovery that glomerular glutaminolysis was impaired in DKD, as indicated by increased Gln concentration and decreased Glu. This result is consistent with evidence from a clinical study in which urinary Gln metabolites were significantly reduced in DKD patients based on metabolomic analysis.^29^ Collectively, these studies suggest that the process of Gln catalyzation and utilization plays a vital role in maintaining podocyte function and metabolic adaptation under DKD, an important question remaining is how the glutaminolysis pathway is blocked.

In humans, two forms of glutaminase (GLS) are designated GLS1 and GLS2.^30^ GLS2 is a rate‐limiting enzyme that catalyses the conversion of Gln into Glu in mitochondria.^31^ Previous studies indicated that HG treatment of podocytes significantly decreased the expression of Gln catabolism‐related enzymes.^32^ In this study, we found that GLS2 expression was downregulated in diabetic podocytes, and GLS2‐mediated Gln metabolic pathway was impaired in glomeruli of DKD mice. In general, GLS2 acts as a mitochondrial energy promoter, as evidenced by the downregulation of GLS2 was negatively correlated with glutaminolysis.^33^ In line with previous reports, our findings suggested that GLS2 downregulation caused by diabetes could inhibit ATP production in podocytes. However, the regulatory mechanism of GLS2 expression in podocytes remains unclear. In this study, we also found that the expression of podocyte nuclear transcription factor LRH‐1 was downregulated in diabetic podocytes. Similarly, previous studies in patients or animal models with diabetes and nonalcoholic fatty liver disease (NAFLD) have found downregulation of LRH‐1 expression in islets and the liver.^34^, ^35^ Additionally, evidence has shown that LRH‐1 plays a role in regulating mitochondrial dynamics and a multitude of metabolic processes.^36^ Here, we demonstrated that the expression of GLS2 in podocytes is regulated by LRH‐1, and the activation or overexpression of LRH‐1 can promote GLS2 expression and restore mitochondrial function, thereby reducing podocyte apoptosis, finally alleviating DKD‐induced renal injury. Consistent with our study, the latest results identified five putative LRH‐1 response elements within the promoter region of GLS2, providing evidence that LRH‐1 was involved in the transcriptional regulation of GLS2.^15^, ^37^ Notably, GLS2 has been recently identified as a transcriptional target of p53, and its expression is responsible for p53‐mediated transcriptional regulation and cell cycle progression.^38^ Interestingly, studies have found that LRH‐1 regulates the recruitment and dissociation of p53 from the DNA.^15^ These results suggest that, in addition to the direct regulation by LRH‐1, LRH‐1 may also regulate the transcription and expression of GLS2 through p53‐mediated pathway. Moreover, the application of LRH‐1 agonist was confirmed to protect against diabetes by repressing inflammation and apoptosis, regulating metabolic homeostasis in pancreatic islets and the liver.^13^, ^19^, ^39^ In contrast, recent studies showed that LRH‐1 knockdown decreased mitochondrial abundance, significantly reduced expression of genes involved in mitochondrial biogenesis and β‐oxidation, and impaired ATP production in hepatocytes and macrophages.^37^, ^40^ It should be noted that our study focused on glutaminolysis and only showed that changes in GLS2 expression regulated by LRH‐1 were associated with the dysregulation of podocyte mitochondrial metabolism in DKD. Because multiple pathways interact to maintain Gln homeostasis, future studies including detailed metabolic flux assays, will be necessary to clarify this in more cell types.

In conclusion, this study unveiled that dysfunction of Gln utilization in podocytes is closely related to abnormal energy metabolism and renal function during DKD. LRH‐1 activation modulates diabetes‐induced podocyte injury by restoring GLS2‐derived glutaminolysis. These results further suggest that LRH‐1 may be a critical regulatory target of energy metabolism in podocytes.

## AUTHOR CONTRIBUTIONS

Jijia Hu and Zongwei Zhang conceived and designed the experiments and wrote the manuscript. Zongwei Zhang and Hongtu Hu performed the main experiments, analysed the data. Keju Yang and Qian Yang participated in some experiments. Wei Liang revised the manuscript. All authors have read and approved the final manuscript.

## FUNDING INFORMATION

The present study was supported by the National Natural Science Foundation of China (8197063 to Wei Liang); Natural Science Foundation of Hubei Province (2022CFB667 to Jijia Hu).

## CONFLICT OF INTEREST STATEMENT

The authors declare that there is no conflict of interest.

## Supporting information


**Figure S1.** LRH‐1 plasmid transfection promotes GLS2 expression. HPCs were transfected with LRH‐1 pcDNA plasmid or vehicle control. (A) Western blot assay showing expression of LRH‐1 and GLS2 among different groups. (B,C) Quantitative analyses of Western blot assay. *p < 0.05 (n = 3).
**Figure S2.** Silencing GLS2 exhibits no specificity on LRH‐1 expression. HPCs were transfected with transfected GLS2 siRNA or scrambled siRNA, and then were incubated with a high concentration (30 mM) of glucose for 24 h. (A) Western blot assay showing expression of LRH‐1 and GLS2 among different groups. (B,C) Quantitative analyses of Western blot assay. *p < 0.05 (*n* = 3); ns, nonsignificance.

## Data Availability

Data supporting the findings of this study are available from the corresponding author upon reasonable request.
