# Supplementary material for: LRH‐1 activation alleviates diabetes‐induced podocyte injury by promoting GLS2‐mediated glutaminolysis
Source: Cell Prolif. 2023 Apr 13;56(11):e13479. doi: 10.1111/cpr.13479 (PMC10623971; doi:10.1111/cpr.13479)
Supplement: Supplementary file 1 — Figure S1. LRH‐1 plasmid transfection promotes GLS2 expression. HPCs were transfected with LRH‐1 pcDNA plasmid or vehicle control. (A) Western blot assay showing expression of LRH‐1 and GLS2 among different groups. (B,C) Quantitative analyses of Western blot assay. *p < 0.05 (n = 3). Figure S2. Silencing GLS2 exhibits no specificity on LRH‐1 expression. HPCs were transfected with transfected GLS2 siRNA or scrambled siRNA, and then were incubated with a high concentration (30 mM) of glucose for 24 h. (A) Western blot assay showing expression of LRH‐1 and GLS2 among different groups. (B,C) Quantitative analyses of Western blot assay. *p < 0.05 (n = 3); ns, nonsignificance. [file CPR-56-e13479-s001.docx]

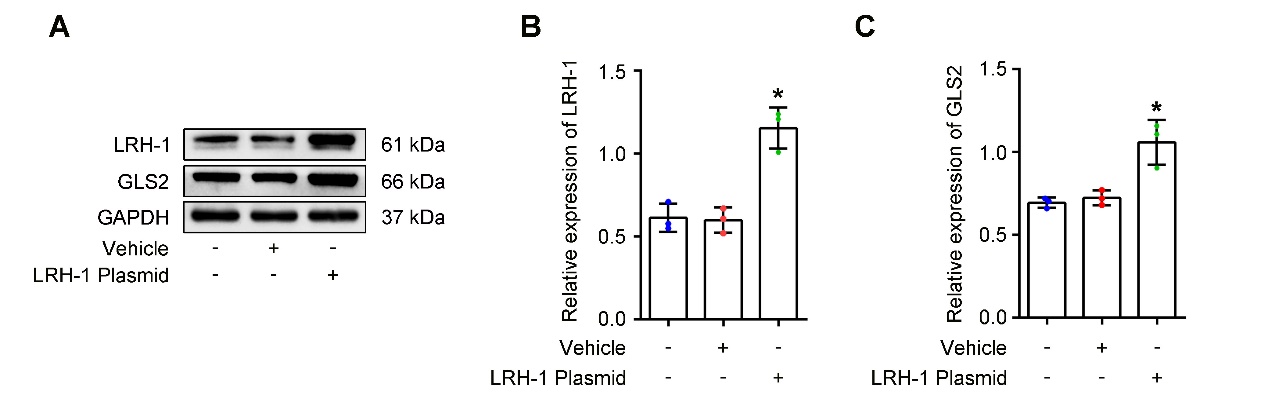


**Supplementary, Figure S1.** LRH-1 plasmid transfection promotes GLS2 expression. HPCs were transfected with LRH-1 pcDNA plasmid or vehicle control. (A) Western blot assay showing expression of LRH-1 and GLS2 among different groups. (B-C) Quantitative analyses of Western blot assay. * p < 0.05 (n = 3).


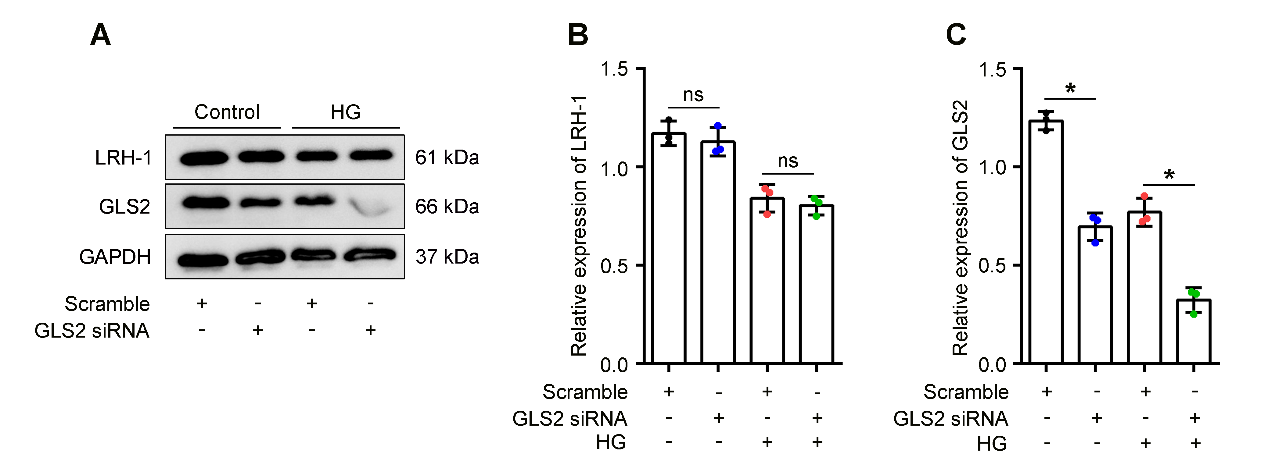
 **Supplementary, Figure S2.** Silencing GLS2 exhibits no specificity on LRH-1 expression. HPCs were transfected with transfected GLS2 siRNA or scrambled siRNA, and then were incubated with a high concentration (30 mM) of glucose for 24 h. (A) Western blot assay showing expression of LRH-1 and GLS2 among different groups. (B-C) Quantitative analyses of Western blot assay. * p < 0.05 (n = 3); ns: nonsignificance.
